# Cases of Practice

**Published:** 1858

**Authors:** D. L. McGugin

**Affiliations:** Prof. of Physiology, Pathology, &c., College of Physicians and Surgeons, Iowa University


### Report of Cases.—Continued.

BY DR. M'GUGIN, A. M., M. D.,

(Prof. of Physiology, Pathology, &c., College of Physicians and Surgeons, Iowa University.)

### *Eczema.*

A very severe case of *eczema rubrum* came under my observation in the Spring of 1855, and then, again, in the same season of 1856. The subject was a little girl, four years old, at the time of the first siezure, and of strumous habit. It appeared simultaneously on different parts of the body, then, afterward, extended from these, until almost the entire surface was covered. The tingling pain and heat of the parts affected was very great; and to this, after a time, succeeded intolerable itching. The condition of the little sufferer was truly pitiable, and appealed most eloquently for relief.

She had, during the Winter, a good appetite, digestion was well performed, nutritive assimilation very active, so much so that she had become full and plethoric. She had indulged in animal food freely, the fatty portions having been prefered. She also used butter freely, as well as highly seasoned gravies, of various combinations. She had enjoyed very little exercise in the open air, but was kept in a warm room during the Winter. When the intense cold had yielded to the milder and more genial weather of the succeeding Spring, the eruption made its appearance, thickly bestudded with minute and transparent vesicles. After a few days these discharged their contents, which, from its acrimonious character, added largely to the existing irritation of the inflamed surface. There was at first a dry cough, with some hoarseness of voice. The tongue was red, and so, also, were the fauces. There was thirst, also, with some arterial excitement. The eyes were red and the lacrymal secretions deficient.

Similar symptoms occurred upon the second attack, in the Spring of 1856, and were preceded by similar circumstances, with regard to confinement during the Winter and the dietetic indulgences.

The treatment pursued was saline aperients, and the tepid bath during the stage of excitement. After this was subdued, the tepid bath of milk and water was continued, and the wonderous glycerine was applied to the surface and proved eminently useful as a soothing application. At the same time the decoction of dulcamara, in which was dissolved the hyd. potassa, in appropriate quantities, three times a day —two hours before or three hours after each meal. Farinaceous diet was strictly enforced and observed, and in two weeks the eruption began to decline.

The rich, oily food, so long indulged in, without the requisite quantity of fresh air for its combustion, resulted in enriching the blood. Nor was there a sufficient amount of exercise to quicken the respiration, or encourage the secretions to the full performance of their functións. Consequently the excretions were deficient, and depurations from the blood imperfectly performed.

These errors were avoided during the past Winter, for she was kept out in the fresh air, and oleaginous food excluded from her diet. The symptoms did not appear.

My experience in the use of the dulcumara, in eczema and other cutaneous eruptions, inclines me to a decided preference for it, as an alterative, over sarsaparilla or any other of the class usually prescribed. The addition of the hyd. potassa increases its properties, and constitutes a very reliable prescription. The stems and leaves, as prepared by the "Shakers," and which is for sale in our drug establishments, will be found pure and reliable.

---

*Intermittent Neuralgia of the Stomach.*

This case had continued for some months regularly to return, with most excrutiating suffering to the individual, and awakening afresh, with each recurrence of the paroxysm, the deepest solicitude for his safety. During this time I had not been consulted in the case, but after a time was requested to see him. I found him suffering much and delirous occasionally. There was fever, with hot skin, dry and furred tongue, bowels constipated, icterode aspect, scanty urine, tenderness over the stomach and the liver and fullness in the colon over the sigmoid flexure. There was much restlessness and anxiety with thirst.

Upon a close examination of the case, I pronounced it to be of malarious origin—a fact which had escaped the notice of his previous attendants. It was found to recur every fourteen days, and such, too, *had* been the periodical characteristics since the first paroxysm—a feature which we should expect would not have escaped notice. The bowels having been freely moved, large doses of quinine with sulph. morphia speedily overcame the paroxysm, after which we entered upon measures to prevent the recurrence of another paroxysm. The following prescription was adopted:

℞ Ext. Cinchonu drachm iss
Ext. Humulus lup drachm ij
Sulph. Quinine scruple ij
Ext. Taraxacum drachm i
Aromat. Sulph. Acid f. drachm ss
Aqua Cinnamon f. ounce vj
M.

Sig. One dessert spoonful three times a day.

This treatment was persisted in and the paroxysms did not return for more than three years, during which the individual enjoyed excellent health.

The above preparation will be found a most valuable prescription in ordinary intermittents, where the paroxysms have been overcome, but prove to return. I have not yet seen a return of the paroxysms in any case after they were once subdued, where the above had been perseveringly used and no undue exposure had occured.

It is respectfully suggested to the consideration of the profession, in malarious localities, in order that the *oprobrium* of being unable to *prevent* the recurrence of the paroxysms, (although it is admitted that we can subdue them for a time,) may be removed.

---

*Menorrhagia.*

The following I extract from the reports of cases in the "Charity Hospital," New Orleans, contained in the "*New Orleans Medical News and Hospital Gazette*," and I do so for two reasons, first, because the case is an interesting one, and the inquiry important; and, second, because I shall append one equally interesting, and may answer the inquiry propounded in the report.

"Service of Dr. W. Brickell. May 22nd, 1857, Mary S. entered ward 35, Charity Hospital, after the regular morning visit. Patient is a native of Germany, æt 38 years—in America 16 years; is rather a short, but well built woman; does general house-work; has never been seriously sick until within the past five months. Seventeen years ago she was first married, lived with her husband ten years, became pregnant one month after marriage, carried fœtus five months and aborted, and has never been pregnant since. She married, a second time, eighteen months after the death of her first husband, and the second husband is still alive. Five months ago she was working hard at the wash-tub, and was a good deal exposed to very cold and inclement weather; her catamenia appeared at the regular time, but were attended with more pain than usual, and came in greater quantities than she had been accustomed to. From that day to this, (May 23d,) she has been the subject of constant menorrhagia, with the single exception of one interval of fourteen days within the past six weeks. The flow of blood has been constant, night and day, and at times profuse; coagula have been formed and thrown off in profusion, but she has never suffered much pain in the uterine region. Uterine contractions have never been immoderate, and never occurred except to expel coagula.

"Condition on 23d of May.—Exceedingly pale, very feeble, but rather cheerful. No fever, yet with pulse indicative of irritability. The functions of the various organs seemed unimpaired; no constipation nor torpor of liver, etc. There being no really urgent symptoms, it was determined to let her lie quietly for twenty-four hours, and watch her symptoms. No vaginal exploration was made, as things were not prepared for the same. External pressure, etc. elicited moderate pain in the pubic region.

"May 24th.—Patient in *statu quo*. Hemorrhage continues in spite of horizontal position, but no other disagreeable symptom has manifested itself. Examination now, by means of a speculum, revealed a perfectly healthy vagina, os and cervix uteri, as far as the latter could be penetrated. The os was not even patulous. The vagina contained about a gill of old coagula, and as soon as the os uteri was exposed to view, several coagula were actively squeezed from it, and then followed a free and continuous stream of pure blood, which coagulated firmly as soon as it fell into the speculum. The introduction of Simpson's Sound into the uterus was now attempted, but, notwithstanding it was faithfully and most carefully tried, the instrument could not be insinuated further than a quarter of an inch. The cervix seemed firmly contracted, and wholly indisposed to yield. Examination by the touch, (per vaginam,) discovered the uterus to be considerably heavier than natural, though pressure against the organ produced no pain. It was determined to put the patient on the use of general remedies. Suffice it to say, that from this time until the 8th of June, she went through the ordinary routine of acetate of lead, ergot, etc. but without the slightest benefit. In fact, at this latter date, (June 8th,) she is a great deal worse. She has continued to bleed, until really her condition is alarming. She is very feeble indeed; her legs are beginning to be puffy, and she appears to be perfectly blanched; there appears to be no red blood in her. Repeated attempts have been made to introduce sounds and bougies into the womb, but never for one moment has the cervix relaxed its spasmodic hold. It seems strange to see coagula leaping, as it were, from the os into the speculum, and yet, when the sound is passed into the os, it is found impossible to urge it through the cervix. The woman's life seems now seriously threatened, and yet all efforts to check the hemorrhage are unavailing.

"On the 11th of June, the patient seeming to be almost in desperate condition, it was resolved to try once more the introduction of a sponge tent into the cervix. The same spasmodic contraction was found, but it was now determined to test the virtues of a solution of atropine to the parts. Through the speculum the solution was poured, *ad libitum*, and held in its proper place. In twenty minutes a sponge tent was partially introduced and allowed to remain. It should be mentioned here, also, that now the introduction and expansion of the speculum gave considerable pain, and, although the os and cervix looked healthy, examination by the rectum discovered considerable and irregular enlargement of the body of the uterus, with pain on pressure.

"After the partial introduction of the tent, she was ordered pretty full doses of Dover's Powders, with beef tea, etc.

"June 12th.—Patient more comfortable; no bleeding, no inconvenience from the tent. The speculum was reapplied and the tent withdrawn. A little blood followed after it, but no coagula. Another tent was applied, the Dover's Powder repeated, and the patient ordered to be quiet.

"June 15th.—By every day repeating the tents, at the same time enlarging them at each application, the whole cervix has been completely dilated, so that the thumb might readily be introduced into the uterus. Strange to say, the patient has improved steadily from the time of the partial introduction of the first one, and now looks and feels better than she has done since she came into the ward. *All hemorrhage has ceased,* her appetite is returning, and she is cheerful.

"Monday, August 10.—Since the 15th of June the patient has had but one slight attack of hemorrhage (20th of June,) and has improved steadily, until now she is discharged apparently perfectly well. She has taken no medicine, and was only required for several weeks to maintain the horizontal position and eat nourishing food.

"What was the cause of hemorrhage in this case? Had the dilation of the cervix any agency in checking the hemorrhage? If so, in what way is it to be explained?"

The above I will follow with the report of a case which I was required to treat:

Mrs. —— aged 35, of leuco-phlegmatic habit, was brought to me from the interior for advice. She was anæmic, pulse 110 and quick, skin, lips, gums and tongue pale and exsanguine. For some time she had suffered from uterine hemorrhage, more or less abundant, but constant, which was believed to be the result of the pressure of a polypus.

At this time the discharge was but small in amount, but pale and watery.

The touch revealed no increase in the size of a normal uterus, and nothing in this way was detected, save a rigidity of one of the lips of the os. The finger, when withdrawn, was covered with poor blood, and the linen showed but a feeble stain. Not fully satisfied with the results of this exploration, the speculum was resorted to, in the hope of detecting the source of this continued drain.

The anterior lip showed a loss of structure, and the sound enabled us to turn it out, which done, there was revealed an ulcer, in depth sufficient to bury the hemisphere of a pea, and in circumference about equal.

From the ulcerated surface there issued, *drop by drop*, this pale, poor blood, which had been gradually and continually draining away her vital forces.

A small tent, saturated, if I may use the term, with the iodide of zinc, dry, was introduced and retained for the next twenty-four hours, at the end of which time it was removed and another, qualified as above, introduced. After the removal of the second tent no blood flowed, and the ulcer healed in a short time. Complete recovery ensued in due time, under the use of appropriate tonics and nutrition.

May not the case of Dr. Brickell, as related above, have been a similar one, with this exception, that the ulceration was higher up and beyond the reach of the eye?

These cases serve to admonish us not to attribute all hemorrhages which flow from the vagina, to the interior of the uterus, and, also, that constant and continued bleeding may issue from an ulcerated surface, when no polypus is present.

Much has been said against the frequent use of the speculum, but in these cases its value was very apparent.

---

### *Adherent Placenta.*

I was requested to attend a lady in parturition, whose youngest child was 17 years old. During gestation with the former one, she suffered from pain in the abdomen to the left side rather, and in the labor which followed, in due time it was found that the placenta was adhered, but to what extent I had no means of knowing, as the patient only knew from the statement of her attending physician at the time that such was the difficulty.

During gestation, in this last pregnancy, she again complained of this pain, and referred it to the other side; but as she arrived in this place but a short time before her confinement she only reported it as having existed, and before labor set in expressed fears of a like result.

The labor went on, however, regularly and naturally, and the child when expelled was large and vigorous. The umbilical cord, however, was very small, and wound around the neck of the child twice. With much difficulty one fold was removed, and even when this was unfolded it was manifestly much shorter than usual so much so that when divided it did not extend beyond the external organs more than three inches.

After the child was born effort was made by friction, grasping, and compression, as well as by the sudden appplication of the hand, the temperature of which was lowered by immersion in ice-water for a time, to induce the uterus to contract, but without producing any appreciable effect. Ergot was given and the friction and compression continued. Cold iced-water was also applied freely and yet no contraction, but very soon an alarming hemorrhage came on embarassing the case still more and endangering the life of the patient.

It was now time to enquire into the situation of the placenta and to my dismay I found its uterine surface almost entirely attached. A small portion around the external boundary was detatched and loose and from broken loops of vessels, the blood was pouring out. Strange to say the parietal walls of the uterus were relaxed and flabby notwithstanding the presence of my hand. The umbilical cord could not be used with which to make traction, as it was almost entirely detached from the placenta, by the force of the pain, when around the neck of the child, in the final expulsive effort. I could only, therefore, use it as a guide, when exploring the location of the placenta, which was attatched to the fundus, and rather to the right side. As there were numerous vessels pouring in their flood tide, and as, too, we were wanting in the natural hæmostatic—the contraction of the uterine fibres, the flooding continued freely. An immediate separation of the placenta was undertaken, but was rendered very difficult by the firmness of the adhesion on the one hand, and the relaxed and unresisting walls of the uterus on the other. An effort at traction along the edge of the placental mass, would bring down the uterus with it, so that in separating it, it was necessary to fix the uterine walls with the backs of the fingers, while the separation was being made with their points. Every portion separated but added to the volume of the discharging blood, and the condition of the patient but too plainly indicated the amount already lost, and the danger of a further considerable expenditure. The feeble efforts of a frightened assistant failed, by external measures, to awaken one particle of contraction, and, therefore, as an immediate separation promised most for my patient, my best efforts were exerted to accomplish that end, which, fortunately, were soon successful. By this time, my associate, Dr. Letcher, accompanied by Dr. Allen, arrived, and all exerted our efforts for her resuscitation. Dr. Letcher succeeded, by constant effort, and by all the means usually resorted to, to induce the uterus to contract, but could get but a feeble effort. This, however, would only prove efficient so long as he continued his efforts, and it was only after an hour that full contraction was induced.

The case was one of unusual embarrassment, for reasons already detailed, which may have been found in the extent and firmness of the adhesion, in the relaxed condition of the uterine walls, in the copiousness of the hemorrhage, and, last and most important, perhaps, in the non-contractility of the uterine fibre.

After a struggle the lady recovered and is now doing well.

Doubtless there had been placentitis at the period when pain was felt as above indicated. The antiphlogistic measures would then have been highly proper, if resorted to in time to have prevented adhesion.

Since then I was called to another case, in charge of another gentleman, in which similar symptoms existed in the last months of gestation.

The lady was of full habit, and although the adhesion was not extensive, it was, nevertheless, very firm. But the uterus was well contracted, so much so as to partially constitute hourglass contraction.

These cases teach the necessity of instituting close inquiry into the condition of a woman in pregnancy, when she complains of pain in the region of the uterus, and if there are inflammatory symptoms they should be controlled if possible. Our suspicions of placentitis may be confirmed, to some exrent, if we find, by auscultatory inquiries, the placental souffle in the locality of the pain. If the woman is of plethoric habit, the necessity for inquiry and interference will be augmented, as in both these cases there was fullness of habit, or plethora.

